# Summary of Foreign and Domestic Medical Journals

**Published:** 1865-10

**Authors:** Charles Nichell


### Summary.

### Summary of Foreign and Domestic Medical Journals.

PREPARED BY CHARLES NICHELL.

J. L. Clarke, F. R. S., on the Pathology of Tetanus, (*London Lancet,*) describes the condition of the spinal cord, in six cases of tetanus. In every one of these there was not only more or less congestion of the blood-vessels, but there were also definite and frequently extensive lesions of structure, such as has never yet been discovered. These lesions consisted of disintegrations of tissue in different stages of progress, from a state of mere softening to that of perfect fluidity, and were accompanied by certain exudations and extensive effusions of blood. They were found chiefly in the grey substance, which, moreover, was in many cases strangely altered in shape—unsymmetrical on the opposite sides, or partially fused with the adjacent white column in a common softened mass. Although lesions of this kind existed in one form or other, in every

*region* of the cord, they were absent in some places, nor did they ever, for a long time together, maintain the same shape, size or appearance, but were constantly and alternately increasing, diminishing or disappearing, at short but variable intervals.

These lesions in tetanus are precisely similar in character to those which the author has discovered in the spinal cords of many ordinary cases of paralysis; and on comparing together the lesions and symptoms of both kinds of diseases, he finds good ground for the support of the following conclusions:

1st—That the lesions are not present, or are present only in a slight degree, in those cases of tetanus which recover.

2d—That they are not the *effects* of the *great functional activity of the cord,* manifested in the violent spasms, but are the effects of a morbid state of the blood-vessels.

3d—That they are not alone the *causes* of the tetanic spasms.

4th—That the tetanic spasms depend on *two separate causes*—firstly, on a *morbidly excitable condition of the grey substance of the cord,* induced by the hyperæmic and morbid state of the blood-vessels, propagated from the injured nerves, and resulting in exudations and disintegrations of tissue; and secondly on *irritation* propagated and spread through the morbidly excitable cord from the same source—from the periphery, by the diseased nerves.

E. H. Greenhow, M. D., in his clinical lectures on diphtheria, (*Medical News and Library*), says that there are two principal varieties of this formidable complaint—namely, that form in which the urgency of the case is due to the local manifestations of the disease, and that other form in which the danger arises from the general constitutional affection. The former of these is especially characterized by the existence of symptoms of apnœa, and the pressing danger is caused by the more or less complete occlusion of the air passages by the membraniform exudation. The latter, on the other hand, is characterized by the predominance of symptoms of asthenia caused by the intensity of the general disease, and the danger to be apprehended is the gradual exhaustion of the vital powers. Although these two forms of diphtheria are so diverse in their more salient characters, and the kinds of danger which attend them, there is yet no doubt of their being only varieties of one and the same disease, for they not only occur constantly during the same epidemic, but very often also in the same household, at or about the same time, and occasionally cases occur which partake more or less of the characters of both forms, usually, however, one of the two classes of symptoms predominate.

Although these two classes of diphtheria are the most important, they are by no means the only form of the disease. In every epidemic there are many cases in which neither the air passages are involved, nor are there any urgent symptoms of general constitutional affections, so that many of these, at any other time, would merely be regarded as cases of common inflammatory sore-throat. Again, cases may present themselves characterized by more or less diphtheric exudation in the fauces, but unattended by urgent symptoms. Such cases usually recover under any rational mode of treatment, and it is the consequent great apparent success in treating cases of such mild symptoms which has sometimes led practitioners to promulgate as a specific for diphtheria some medicine with which they have treated large numbers of them; the truth being that by far the greater proportion of these cases required only common care, and would probably have recovered without any medical treatment at all. But although these mild cases so often do well without any or no particular medicine, still caution is necessary against neglecting them, as even the mildest cases require careful watching, because either by an invasion of the air passages or by the accession of constitutional symptoms a case which in its first stages appeared of the mildest kind may subsequently assume a most serious form.

Charles Hunter, Esq., on the hypodermic administration of certain medicines. Mr. Hunter proposes the injection of medicines into the cellular tissue with their general therapeutic object in view. In the case of medicines thus injected for their general affects, he calls the method the "hypodermic" to distinguish it from the endermic and from the local injection of Wood. From the endermic method it differs much, it being a superficial application, which must be uncertain in its action, and which may act powerfully and dangerously or prove wholly useless. The hypodermic differs from the method of Wood, the latter plan having for its object the local treatment of local affections. The injection was supposed by Dr. Wood to be efficacious simply through the localization. Theoretically this method must be limited in its sphere of action to neuralgia or sciatica; to those cases alone accessible to the point of the injecting syringe. Mr. Hunter, in advancing the hypodermic method, maintained that localization of the injection in neuralgic cases, was theoretically wrong, and practically unnecessary. In support of this he advances the following propositions:

1.—That equal effects followed distant and local injections in neuralgic cases.

2.—That by distant injections the ill-effects of repeated localization were avoided, such as local irritation, thickening of the skin, abscesses, etc.

3.—That diseases can be treated with benefit and curatively by this plan, which are neither local nor neuralgic, and which have failed to receive benefit from other modes of medicinal administration.

The hypodermic method is considered by the author superior to the stomachic, rectal and endermic modes of medicinal administrations in emergent cases, in which the indications are for anodynes, anti-spasmodics, hypnotics, and nerve-tonics. And he has found greater and more permanent benefit to accrue from this mode of treatment than from the stomachic use of morphia, atropia, codeia, and other alkaloids. Many hours often elapse before any benefit follows the use of stomachic medication, but by the injection of the cellular tissue the desired relief can be obtained in from five to thirty minutes, instead of after many hours.

Report upon the Epidemic occurring at "Maplewood Young Ladies' Institute," Pittsfield, Mass., in July and August, 1864, by Drs. A. B. Palmer, C. L. Ford and Pliny Earle. *(Boston Medical and Surgical Journal.)* Maplewood Young Ladies' Institute, a well-established and popular boarding school, at Pittsfield, Mass., has lately been visited by a violent outbreak of sickness, bringing prominently before the medical profession and the public, various questions connected with its sanitary condition.

During the later parts of July, 1864—from the 23d to the 29th—five persons were attacked with a severe form of disease, and up to the 10th of August some thirty others were sufficiently indisposed to require the advice of physicians. There were at the time seventy-seven young ladies who were boarding, lodging and receiving instruction in the institution. Besides the principal and his immediate family, seven or eight teachers resided in the establishment. The whole family, including servants, consisted of about one hundred and twelve persons.

Of the five persons taken seriously ill, as before mentioned, one was a teacher, who recovered after a long and severe sickness; but the other four, three of them pupils, and the other a servant girl, died.

At the close of the term, on the 10th of August, the pupils hastily dispersed, many of them being so seriously indisposed at the time of leaving, that they performed their journeys home under the influence of quinine and alcoholic stimulants; while one, too ill to leave, remained sick for some time in the institute, and two others proceeded no further than a private house in the village, where they each suffered from a severe and protracted illness, but ultimately recovered. A week after the close of term, two young ladies, day scholars, residing in the village, were attacked in a manner similar to the others. Three servants were also attacked at the same time, one of which died, and numerous reports of sickness and deaths among those who had left were shortly afterwards received.

The large proportion of the pupils reported to have been attacked and the unusual severity and fatality of the cases, pointed to some local cause at the institute. A committee, consisting of Drs. Palmer, Ford and Earle, found upon special inquiries, that of the seventy-seven pupils in attendance, fifty-one have had typhoid fever. Of the remaining not reported as having typhoid fever, nine had, in a milder form, premonitory symptoms, which speedily yielded to treatment; one had dysentery; one reported slow fever, one anæmia, two unwell; the remaining considering themselves as "well." Of the 51 cases of typhoid fever among the pupils 13 terminated fatally, or about 25.5 per cent. The remainder having more or less recovered.

From the investigations of the committee it appears that the closet vaults have been shallow, and filled nearly to the surface of the ground with semi-fluid materials. A few rods from the building was situated, until removed last July, a barn which had been there for many years, and beyond it a barn-yard containing water in which the swine were reported to have wallowed. The odors emitted from these sources, it is stated to have been such that not unfrequently windows were obliged to be closed, particularly at night, to prevent the influx of the outer air, as the confined atmosphere within the rooms was less offensive than the air from without. The conclusions to which the foregoing facts so strongly tend are, that the epidemic of typhoid fever at the Maplewood Institute, was caused by the malarial effluvia arising from these accumulations.

